# Non-Invasive Prediction of Hepatic Fibrosis in Patients With Chronic HCV Based on the Routine Pre-Treatment Workup

**DOI:** 10.5812/hepatmon.6718

**Published:** 2012-11-01

**Authors:** Marwa Khairy, Mahassen Abdel-Rahman, Maissa El-Raziky, Wafaa El-Akel, Naglaa Zayed, Hany Khatab, Gamal Esmat

**Affiliations:** 1 Endemic Medicine and Hepatology Department, Faculty of Medicine, Cairo University, Cairo, Egypt; 2 Pathology Department, Faculty of Medicine, Cairo University, Cairo, Egypt

**Keywords:** Hepatitis C, Liver Cirrhosis, Fibrosis

## Abstract

**Background:**

Hepatic fibrosis is an inclusion indicator for treatment and a major independent predictor of treatment response in patients with chronic hepatitis C. Liver biopsy, considered as the “gold standard” for evaluating liver fibrosis, has carried some drawbacks. Currently used noninvasive predictors of fibrosis are considered less accurate than liver biopsy.

**Objectives:**

Our aim was to assess noninvasive predictors of fibrosis in patients with chronic hepatitis C using the routine laboratory pre-treatment workup.

**Patients and Methods:**

Cross sectional study including 4289 Egyptian patients with chronic hepatitis C were assessed for the need to interferon and ribavirin therapy. Routine pre-treatment workup and reference needle liver biopsy were performed. FIB-4 index, APRI and modified APRI scores were validated. Patients were divided into two groups, first with no or minimal fibrosis, and second with moderate and marked fibrosis using the Metavir score.

**Results:**

Multivariate logistic regression analysis showed that age, body mass index, aspartate aminotransferase, alpha fetoprotein, platelets count, FIB-4 index, APRI and modified APRI score were significant independent predictors of fibrosis. Age > 43 years, aspartate aminotransferase > 47U/L, platelets < 205×103/mm^3^, and alpha fetoprotein > 2.6 ng/ml had the highest cutoff points in receiver operator characteristic curves. Taking into account the four variables together; the presence of ≥ 2 variables is associated with moderate and advanced fibrosis with a sensitivity of 0.81, specificity of 0.5, positive predictive value of 0.53 and negative predictive value of 0.79. FIB-4 index represented the best performing receiver operator characteristic curve for diagnosing moderate and marked fibrosis among other independent factors with a sensitivity of 0.74, specificity of 0.6, positive predictive value of 0.56 and negative predictive value of 0.76.

**Conclusions:**

Chronic HCV pre-treatment routine work up and composite fibrosis scores are good noninvasive predictor of liver fibrosis and can be used as an alternative method to invasive liver biopsy without adding more financial expenses to the treatment.

## 1. Background

Hepatitis C virus (HCV) infection is one of the main causes of chronic liver disease worldwide (1). The long-term hepatic impact of HCV infection is highly variable (2). The number of chronically infected persons worldwide may exceed 200 million (3), with Egypt having the highest prevalence worldwide, 15% of the population had positive results of antibodies tests to HCV whereas 10 % were found to have HCV viraemia (4) In Egypt, HCV is considered as a major cause of chronic hepatitis, liver cirrhosis, HCC and liver transplantation (5). The current recommended regimen for chronic HCV management is the combination of pegylated interferon and adjusted body weight Ribavirin (6). Advanced fibrosis and cirrhosis have been shown to be major independent predictors of non-response to treatment infection (7). Liver biopsy has been considered as the “gold standard” for defining liver disease status, despite the fact it carries some drawbacks - as risk of bleeding, sampling error and inter-observer variability - which have raised questions on its value (8). Thus, alternative noninvasive methods are studied to establish information on the extent of fibrosis by focusing on noninvasive blood marker indicators (9). Several factors have been clearly shown to be associated with fibrosis progression rate: duration of infection, age, male gender, alcohol consumption, HIV co-infection and low CD4 count and metabolic conditions are emerging as independent co-factors of fibrogenesis. Recent validation of non-invasive biomarkers should facilitate the study of fibrosis progression in large populations (10). The objective of this study is to assess hepatic fibrosis non-invasively in patients with chronic HCV using the routine laboratory pre-treatment workup as an alternative to liver biopsy which could be economical, reliable, easy to apply and acceptable by domain experts.

## 2. Objectives

The proposed predictors would minimize the cost and the time of assessing the liver fibrosis stage and also reduce the need for liver biopsy.

## 3. Patients and Methods

### 3.1. Selection of Patients

Retrospective, cross sectional nationwide study focused on interferon-naive patients with chronic HCV including 4289 patients who were examined from Fatemyia Hospital located in Cairo. This center is one of the twenty one referral centers for treatment of HCV in Egypt under the supervision of Ministry of health. This national project in the period from 2007 to 2010 included more than 200.000 Egyptian patients with chronic HCV for treatment of chronic HCV infection with pegylated interferon and ribavirin. Patients were subjected to a thorough history taking, clinical examination and routine pre-treatment laboratory work up including: complete blood count (CBC), serum transaminases (AST, ALT), total and conjugated bilirubin, albumin, alkaline phosphatase, prothrombin time and concentration, urea, creatinine, fasting blood sugar, HBsAg, HBcAb, anti-shistosomal antibodies, anti-nuclear antibodies (ANA), alpha fetoprotein (AFP), thyroid stimulating hormone (TSH), and HCV quantitative PCR.

### 3.2. Histological Classification

Histopathological examination of ultrasound guided percutaneous liver biopsy requires using 16 gauge semi-automated biopsy needles. Liver specimens of 15mm in length with minimal 4 portal tracts were fixed in 10% neutral formalin, then processed and embedded in paraffin. Sections were stained with haematoxylin and eosin, and Masson trichrome stains for detection of fibrosis. Metavir scoring system demonstrated different stages of fibrosis (F0-F4) and grades of necro-inflammatory changes (A0-A3). (11). The histopathological examination of all the subjected liver biopsies was performed by a single hepatology expert pathologist. Patients were divided into two groups based on the degree of fibrosis; group I: patients with no or minimal fibrosis (< F2) and group II: patients with moderate and marked fibrosis (≥ F2).

### 3.3. Calculated Scores

APRI, modified APRI and FIB-4 scores were calculated. The APRI score was calculated using Wai’s formula (12):

(AST/upper limit of normal)/platelet count (expressed as platelets × 109/L) × 100

The modified APRI score was calculated as follow: (13)

[Age (y) x (AST/upper limit of normal] / [Serum albumin (g/dl) x platelet count (expressed as platelets × 109/L) × 100]

The FIB-4 score was calculated using Sterling’s formula (14) as:

Age (y) × AST (IU/l) /platelet count (×109/liter) ×√ALT (IU/l))

### 3.4. Patients Consent

Informed written consent from each patient and local ethical committee approval were obtained before starting the data collection. With respect to patients’ confidentiality, patients were represented in the study by code numbers. All personal data was concealed. The study protocol conformed to the ethical guidelines of the 1975 Declaration of Helsinki as reflected in a priori approval by the institution’s human research committee.

### 3.5. Statistical Analysis

All patients’ data was tabulated and processed using SPSS 10.0 for Windows XP. The quantitative data was described with mean, standard deviation (SD) or range and compared with t-student test. Pearson correlation was conducted to correlate continuous parameters. Multivariate backwards stepwise binary logistic regression analysis with significant fibrosis (F ≥ 2) - as the dependent factor - was performed. ROC (receiver operator characteristic) curve(s) were constructed to assess area under the curve (AUC)), patients were classified into two groups (below and above the cutoff values). Best cutoff values for the independent variables were determined based on the nearest point to top left point in the ROC curve. P value below 0.05 was considered significant. 

## 4. Results

**Figure 1:**
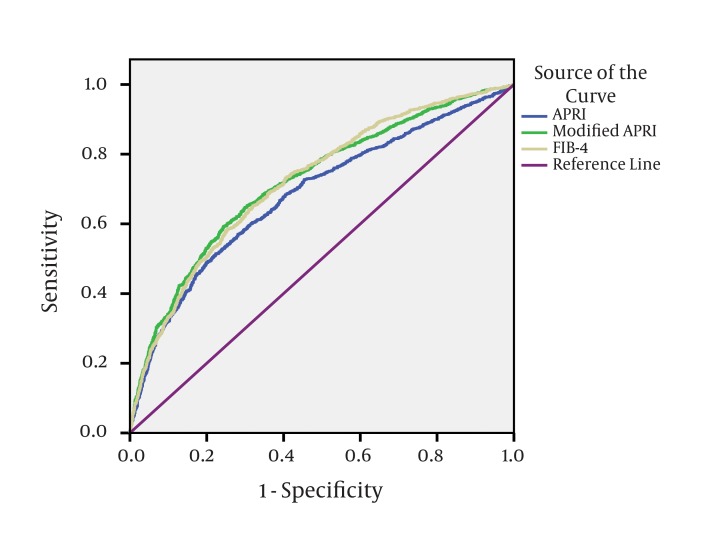
ROC Curve of the APRI, Modified APRI and FIB-4 of the Studied Patients

The demographic criteria of the studied patients showed that the mean age of the patients was 41 years, with older age in patients with moderate and severe fibrosis (r = 0.282, P < 0.01). 81.5% (3495 patients) of the study population were male. Female patients showed significant more advanced hepatic fibrosis than males (P = 0.001) (Table 1). The mean BMI (body mass index) of the studied patients was 28.1 with a significant direct correlation with hepatic fibrosis (r = 0.13, P < 0.01). According to the liver biopsy; group II involving patients ≥ F2 present 41.5% (F2 = 21.1%, F3 = 13.1% and F4 = 7.3%) of the studied patients, while group I including patients with no or minimal fibrosis < F2 represent 58.5% of the patients (F0 = 1.2% and F1 = 57.3%). The necro-inflammatory activity (A) elicited in liver biopsies were associated with progression of liver fibrosis (P = < 0.01) (Table 2). Univariate analysis of the patients’ laboratory data showed that, the serum ALT, AST, bilirubin, fasting blood glucose and AFP were statistically significantly higher in group II, in contrast the serum albumin, prothrombin concentration, hemoglobin, WBCs count, ANC and mean platelet count were significantly lower in group II as shown in Table 3. On the other hand, the HCV PCR count, ANA level, TSH level and anti-schistosomal antibodies level showed no significant association with hepatic fibrosis. The multivariate binary logistic regression analysis showed that the age, BMI, AST, AFP and platelets count are remained as the significant predictor of fibrosis as shown in Table 4. ROC curves for age, AST, platelets and AFP were constructed to determine cutoff points which are more sensitive to detect fibrosis; the cut points were age > 43 years, AST > 47 U/L, platelets < 205x103/mm3, AFP > 2.6 ng/ml. Taking into account four variables together as a single score to get more sensitive way for diagnosing fibrosis, the presence of two or more of these four variables (i.e. having score > 2) is associated with moderate and severe fibrosis (≥ F2) with a sensitivity of 0.81, specificity of 0.5, PPV of 0.53 and NPV of 0.79. Each predictor involved in the score is represented by one point. The percentage of patients involved into the five categories is shown in Table 5. The APRI, modified APRI and the FIB-4 were significant independent predictors of fibrosis (P < 0.01).The APRI, modified APRI and FIB-4 scores were plotted in ROC curves with FIB-4 index having the best performing curve for diagnosing moderate or severe fibrosis with a sensitivity of 0.74, specificity of 0.6, PPV of 0.56 and NPV of 0.76 as in Figure 1.

**Table 1:** Demographic Criteria of the Studied Patients in Relation to Fibrosis

	Group I, No. (%)	Group II, No. (%)	*P *value	Correlation, r
**Gender**			0.001	
Male a	2083 (83.1%)	1412 (79.2%)		
Female b	424 (16.9%)	370 (20.8%)		
**Age, y, Mean ± SD**	39.33 ± 10.02	44.76 ± 8.61	< 0.001	0.282
**BMI, kg/m^2^, Mean ± SD**	27.64 ± 4.22	28.84 ± 4.37	< 0.001	0.133

Abbreviation: BMI, body mass index.

^a^Total No. (%) of males: 3495 (81.5%)

^b^Total No. (%) of females: 794 (18.5%)

**Table 2:** Histological Activity of the Studied Patients in Relation to Fibrosis

	Group I, No. (%)	Group II, No. (%)	*P* value
**A0**	4 (0.2%)	1 (0.1%)	< 0.01
**A1**	1975 (78.8%)	596 (33.4%)	< 0.01
**A2**	466 (18.6%)	941 (52.8%)	< 0.01
**A3**	62 (2.5%)	244 (13.7%)	< 0.01

**Table 3:** Laboratory Data of the Studied Patients in Relation to Fibrosis

	Patients, Mean ± SD	Group I, Mean ± SD	Group II, Mean ± SD	*P *value
** Hemoglobin, g/dl**	14.18 ± 0.02	14.25 ± 1.47	14.09 ± 1.53	0.001
** White blood cells, /mm^3^**	6.53 ± 0.03	6.58 ± 1.79	6.46 ± 1.81	0.022
** ANC, /mm^3^**	3.42 ± 0.02	39.33 ± 10.02	39.33 ± 10.02	< 0.001
** Platelets, /mm^3^**	215.07 ± 0.96	227.87± 61.93	197.02 ± 58.97	< 0.001
** AST (40), IU/ml**	56.74 ± 0.65	49.34 ± 29.59	67.18 ± 53.83	< 0.001
** ALT (40), IU/ml**	63.67 ± 0.64	58.98 ± 38.40	70.28 ± 45.97	< 0.001
** Total Bilirubin, mg/dl**	0.80 ± 0.00	0.78 ± 0.26	0.81 ± 0.28	< 0.001
** Indirect Bilirubin, mg/dl**	0.58 ± 0.00	0.58 ± 0.25	0.58 ± 0.25	0.774
** Albumin, g/dl**	4.22 ± 0.01	4.28 ± 0.46	4.14 ± 0.47	< 0.001
** Prothrombin conc., %**	86.69 ± 0.17	87.97 ± 10.24	84.9 ± 10.62	< 0.001
** Creatinine, mg/dl**	0.90 ± 0.00	0.90 ± 0.20	0.89 ± 0.20	0.144
** Glucose, g/dl**	99.65 ± 0.46	97.67 ± 27.66	102.32 ± 32.33	< 0.001
** AFP (10), ng/dl**	6.32 ± 0.18	6.75 ± 4.26	16.11 ± 9.24	< 0.001
** HCV RNA, IU a, No. (%)**	0.91 (4.1)	0.98 (4.0)	0.81 (4.4)	0.68

Abbreviations: ANC, absolute neutrophilic count; AST, serum aspartate aminotransferase; ALT, serum alanine aminotransferase; AFP, alpha fetoprotein; IU; international unit.

^a^Median (IQR) Mann-Whitney u test.

**Table 4:** Multivariate Logistic Regression Analysis of the Studied Parameters

	OR	SE	*P* value	95% CI
**Age, y**	1.034	0.0054	< 0.001	1.024-1.045
**BMI, kg/m^2^**	1.027	0.0116	0.01	1.004-1.050
**AST, IU/ml**	1.006	0.0014	< 0.001	1.003-1.008
**AFP, ng/dl**	1.035	0.0073	< 0.001	1.020-1.049
**Platelets, /mm^3^**	0.995	0.0008	< 0.001	0.993-0.996

Abbreviations: AST, serum aspartate aminotransferase; AFP, alpha fetoprotein; BMI, body mass index; OR, odd’s ratio; SE, standard of error.

**Table 5:** Prevalence of Fibrosis in Relation to the Performance Score

	Fibrosis Groups		*P *value
	No or Minimal Fibrosis (n = 2507), No. (%)	Moderate or Severe Fibrosis (n = 1782), No. (%)	
**0.00 (n = 567)**	481 (19.2%)	86 (4.8%)	< 0.01
**1.00 (n = 1080)**	798 (31.8%)	282 (15.8%)	< 0.01
**2.00 (n = 1198)**	699 (27.9%)	499 (28.0%)	< 0.01
**3.00 (n = 1001)**	416 (16.6%)	585 (32.8%)	< 0.01
**4.00 (443)**	113 (4.5%)	330 (18.5%)	< 0.01

## 5. Discussion 

Liver fibrosis is considered as a corner stone in decision making to start treatment with pegylated interferon and ribavirin in patients with chronic HCV. Invasive diagnosis using liver biopsy with histological examination is considered as the gold standard for the assessment of fibrosis but hampering several disadvantages as sampling error and inter-observer disagreement. In addition to the patient low adherence, the financial cost of the biopsy including needle cost, preparation of specimen and analysis are adding obstacles. In a country like Egypt, treatment of chronic HCV represents a governmental economic burden in a population having the highest prevalence of chronic HCV worldwide (15-19% of the population) (4). In addition to the therapy costs, the pre-enrollment investigations add to the financial burden especially that not all the investigated patients would be included for the treatment as they would not all fulfill the guidelines inclusion criteria. For all these reasons, development of a non-invasive panel to predict liver fibrosis instate of liver biopsy with almost the same accuracy (sensitivity and specificity) is needed. Liver stiffness measurement using FibroScan (Echosense, Paris, France) (15), serum serogates (16) and biochemical markers such as fibrotest (17) and glycomics (18) can constitute a non-invasive, reproducible and reliable true alternative to liver biopsy. Our study is aimed at using the routine pre-treatment laboratory workup as a non-invasive method to predict fibrosis as well as the validation of APRI, modified APRI and FIB-4 fibrosis predictor scoring to diagnose liver fibrosis without adding additional cost to the treatment burden. The older the age of the studied patients the more the degree of hepatic fibrosis, this could be explained by the longer duration of exposure to HCV infection. The age of acquisition of infection is even more important than duration of the infection, where the rate of fibrosis progression was higher in older patients irrespective of duration of the infection (19, 20). Thus, the early diagnosis (by strong screening program) and treatment of chronic HCV infection is a corner stone in the prevention and progression of hepatic fibrosis and the related complications. While hepatic fibrosis was statistically significant in female gender in the univariate analysis, the multivariate analysis results was insignificantly contrary to previous studies where hepatic fibrosis increased in males compared to females who showed slower progression to cirrhosis than males of the same age (19). Female patients in our study showed a higher degree of hepatic fibrosis which were older with higher BMI compared to male patients. Slow progression of hepatic fibrosis in young females may be contributed to the protective effects of the female sex hormones (estrogen and progesterone) against fibrosis; this role is lost after the menopause leading to progression of fibrosis. The possible inhibitory effect of female hormones such as 17β-estradiol (the most potent physiological estrogen) and progesterone on HCV RNA replication, HCV protein synthesis and production of HCV infectious particles (virions) were analyzed. It was found that estrogen and not progesterone significantly inhibited production of the HCV virion without inhibiting HCV RNA replication or HCV protein synthesis (21, 22). Obesity with BMI > 30 was associated with higher degree of hepatic fibrosis; this might be explained by the hepatic inflammation mediated fibrogenesis in patients with hepatic steatosis. Recent studies showed that in patients with different HCV genotypes, steatosis is confirmed as significantly and independently associated with fibrosis in patients with chronic HCV (23). Our results support the role of serum AST level as a good predictive variable for histological activity and hepatic fibrosis. As previously studied, low AST values reflects less severe histological parameters, extent of hepatic fibrosis, portal inflammation and piecemeal necrosis. Significant positive correlation between AST values and extent of hepatic fibrosis in combination with platelets count (24), age, total cholesterol level, insulin resistance (by homeostasis model), and past alcohol intake were reported (25, 26). The severity of hepatic fibrosis was associated with the significant elevation of serum AFP level in our study. This result comes together with many studies that found higher serum AFP levels were significantly correlated with advanced fibrosis /cirrhosis in chronic HCV (27, 28). Although in univariate analysis the hemoglobin level, white blood count and platelets count were significant, the platelets count remained the only parameter in the blood profile with a significant value in prediction of fibrosis by multivariant logistic regression analysis. The APRI and modified APRI were also significant predictors of fibrosis in the study. These results were in accordance with recent studies showing the APRI score as a significant predictor of fibrosis (29-31). Sterling et al. described the FIB-4 index, which consists of ALT level, AST level, platelets count and age, for assessing fibrosis in a large cohort of patients with HIV/HCV co-infection (14). The FIB-4 index has also been validated as an inexpensive and accurate marker of fibrosis in the context of HCV mono-infection (32). In our results, the FIB-4 has the best performing ROC curve in the diagnosis of moderate and severe fibrosis which comes together with other studies (30, 31). The age of the patients, serum AST level, platelets count and alpha fetoprotein combined together in predictive score of hepatic fibrosis as well as APRI, modified APRI and the FIB-4 were significant predictors of fibrosis. Noninvasive parameters can be used as good predictor of liver fibrosis in chronic hepatitis C which can minimize the need for liver biopsy. The use of these indices is of benefit as they do not add more cost or burden to the patients routine workup of the pre-treatment with pegylated interferon and ribavirin therapy. The future combination of the previously mentioned serum non-invasive predictors of fibrosis and the fibroscan may offer better results and performance which needs to be confirmed. The routine pre-treatment work up of patients with chronic HCV may be helpful to predict fibrosis and also be used as a predictor of treatment response.
